# Combating *Acanthamoeba* spp. cysts: what are the options?

**DOI:** 10.1186/s13071-017-2572-z

**Published:** 2018-01-09

**Authors:** Ayaz Anwar, Naveed Ahmed Khan, Ruqaiyyah Siddiqui

**Affiliations:** Department of Biological Sciences, School of Science and Technology, Sunway University, Subang Jaya, Malaysia

**Keywords:** *Acanthamoeba*, Cyst, Encystation, Excystation, Therapeutic targets

## Abstract

*Acanthamoeba* spp. are protist pathogens and causative agents of serious infections including keratitis and granulomatous amoebic encephalitis. Its ability to convert into dormant and highly resistant cysts form limits effectiveness of available therapeutic agents and presents a pivotal challenge for drug development. During the cyst stage, *Acanthamoeba* is protected by the presence of hardy cyst walls, comprised primarily of carbohydrates and cyst-specific proteins, hence synthesis inhibition and/or degradation of cyst walls is of major interest. This review focuses on targeting of *Acanthamoeba* cysts by identifying viable therapeutic targets.

## Background

**Fig. 1:**
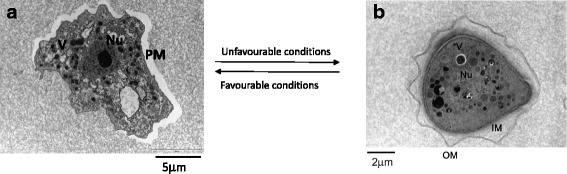
Life cycle of *Acanthamoeba*. Under harsh conditions, *Acanthamoeba* trophozoites transform into a resistant cyst form, while favourable conditions permit vegetative growth. **a** Transmission electron micrograph of trophozoite stage of *Acanthamoeba*. **b** Transmission electron micrograph of *Acanthamoeba* cyst. *Abbreviations*: Nu, nucleus; V, vacuoles; M, mitochondria; PM, plasma membrane; OU, outer membrane; IM, inner membrane

*Acanthamoeba* spp. are opportunistic free living amoebae, commonly found in water and soil. *Acanthamoeba* exist in two forms: (i) an active trophozoite stage and (ii) a resistant double-walled cyst stage (Fig. 1). Encystment usually occurs on exposure of *Acanthamoeba* to harsh conditions such as change in pH, lack of nutrients, treatment with therapeutic agents etc. [1]. *Acanthamoeba* produce two major infections, namely *Acanthamoeba* keratitis (AK), a painful sight-threatening infection, and granulomatous amoebic encephalitis (GAE), a rare but fatal infection of the central nervous system resulting in death in more than 95% of cases [2]. AK is generally associated with contact lens wearers and remains an elusive problem in spite of advances in antimicrobial chemotherapy and eye care [3]. AK is still considered as a rare disease and is usually diagnosed late which makes it harder to treat [4]. The current course of managing *Acanthamoeba* keratitis infections includes a combination of antimicrobial agents such as biguanides and diamidines, while in some cases a topical azole and/or neomycin is added in combination [5]. The use of corticosteroids is controversial as they can cause decline in immunological response of the patients, but some reports show their use can lead to improvement in therapy [6–8]. Based upon the severity of AK and as a last resort, the only viable surgical option is penetrating keratoplasty. Despite advances in combination therapies and surgery, cyst resistance to therapeutic agents and recurrence of infection due to excystment remains a challenge that is yet to be addressed. A major obstacle in overcoming treatment of *Acanthamoeba* infections is the sturdy nature of the cysts and their resistance to physical and chemical damage. Herein, we describe strategies that can be utilised to target *Acanthamoeba* cysts.

## Targeting the cyst wall

**Fig. 2:**
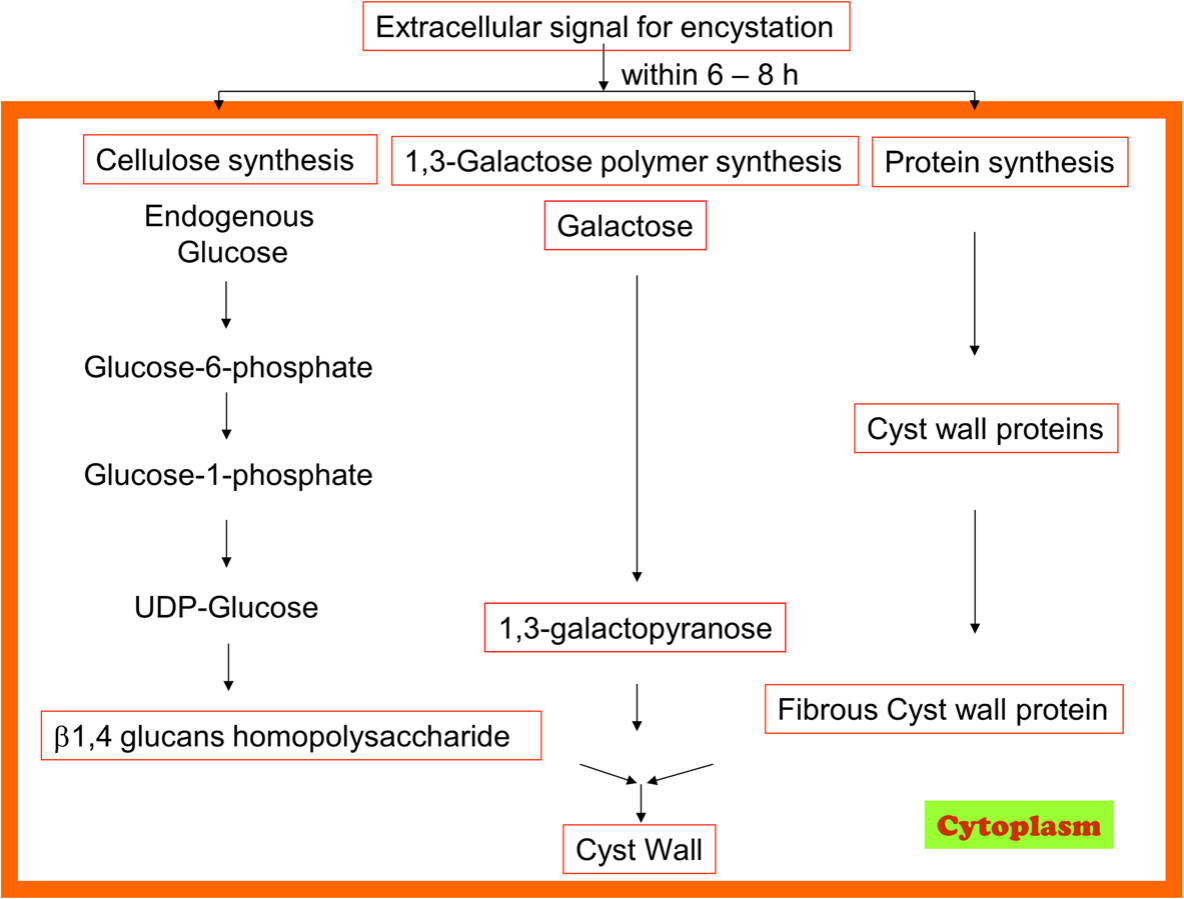
Cyst wall biosynthesis involves polysaccharides and cyst-specific protein synthesis

*Acanthamoeba* cysts are double-walled, consisting of an ectocyst and an endocyst. The ectocyst is formed during the initial stage of encystment and appears as an amorphous, discontinuous layer while the endocyst has a fine granular texture, and is uniformly thicker as compared to the former [9]. As the cyst walls provide a physical barrier for drugs to target amoeba residing within the shell, it is important to understand their biochemistry. Once we understand the basic molecular structure of the hardy shell, it is possible to design strategies to degrade cyst walls and kill the parasite residing within the shell. Using radiolabelling, several studies have been conducted to understand the chemical composition of *Acanthamoeba* cysts, and it is reported that the endocyst consists of polysaccharides, mainly cellulose, while the ectocyst consists of a mixture of proteins and polysaccharides [10, 11]. *Acanthamoeba* cyst walls contain 33% proteins, 4–6% lipids, 35% carbohydrates (mostly cellulose), 8% ash, and 20% unidentified material [10]. Among carbohydrates, cellulose was identified as a major constituent. The precursor of cellulose is glucose that is incorporated into the cell wall as β(1 → 4)-glucans (i.e. cellulose) (Fig. 2) [11]. In contrast, our studies regarding gas chromatography tandem mass spectrometry (GC-MS) based carbohydrate analysis of *Acanthamoeba castellanii* belonging to the T4 genotype identified galactose as a major constituent of the cyst wall, instead of glucose [12]. The carbohydrate components of the cyst wall showed that it contained about 48% galactose and 44% glucose. Furthermore, linkage analysis revealed the presence of 3-linked galactopyranose (1,3-linked galactose) as the highest constituent of the cyst wall (about 29%), while 4-linked glucopyranose (β-1,4-linked glucose, i.e. likely cellulose) was 22% as the second major component [12]. These findings provide novel targets (3-linked galactopyranose) against *Acanthamoeba* cyst walls, in addition to the perceived cellulose (1,4-linked glucose) as the only target to degrade *Acanthamoeba* cysts. The use of enzymatic catalysts that can hydrolyse complex sugars can serve as potential agents to target cyst walls via degradation of specific sugar linkages. Once cyst walls are degraded, it is easier to target *Acanthamoeba*. This concept was proven by our team [13] who showed that targeting *Acanthamoeba* cysts with cellulase enzyme in combination with chlorhexidine can effectively abolish *Acanthamoeba* cyst viability. In contrast, cellulase or chlorhexidine alone had limited effects against *Acanthamoeba* cysts [13]. As the presence of cellulose is limited to plants, fungi, and some bacteria/protists, cellulose-specific drugs should exhibit limited, if any, side effects on host cells. In another report, Lorenzo-Morales et al. [14] established the role of glycogen phosphorylase in encystment as a contributor to glycogen breakdown, and demonstrated its requirement for formation of the endocyst [14]. Hence, utilizing hydrolytic enzymes such as specific glycosidases to do nature’s work in favour of particular application against resistant pathogens holds promise in the development of novel and target-specific therapeutic agents. The advantage of using specific enzymes for targeting particular sugar linkages is of key interest. Based upon chemical composition of the target, one enzyme can be found to be more useful against a given species than the other. For example, endo β-galactofuranosidase obtained from *Bacillus* spp. is specifically shown to degrade β-1-6 galactofuranoside linkages in the polysaccharide of *Fusarium* spp. [15]. Similarly, as *Acanthamoeba* cyst walls contains about 48% galactose [12], targeting 1,3-linked galactose linkage with a specific enzyme may turn out to be a breakthrough in degrading *Acanthamoeba* cysts. Such enzymes of glycoside hydrolase class may serve as potential agents to target cyst walls due to their specificity towards degrading gal-glu and gal-gal linkages. Furthermore, the non-toxicity of these enzymes to mammalian cells makes them an ideal candidate for evaluation of cyst-degrading agents. Among carbohydrate-targeting drugs, research in diabetes has received the most attention. A plethora of drugs are available and/or have been tested for antidiabetic effects that function by metabolizing sugars and/or inhibit their synthesis. As some of the compounds may exhibit broad-spectrum activity, it is reasonable to determine their effects against carbohydrate-linked polymer and/or its synthesis in the cyst walls of *Acanthamoeba*. In particular, as cyst walls of *Acanthamoeba* contain glucose as another major constituent, the glycogen-targeting antidiabetic drugs may be of value in testing against cyst walls synthesis. Although antidiabetic drugs are consisting of a variety of functional agents such as glucosidase inhibitors, dipeptidyl peptidase-4 inhibitors, sulfonyl ureas, insulin, incretin mimetic etc. to name a few, the depletion of cyst walls by deconstruction of sugar linkages or inhibiting polymer synthesis is worth investigating. Sulfonyl ureas such as glimepiride, biguanides such as metformin, glucosidase inhibitors such as acarbose, meglitinides such as glucuronidation agents, DPP4 inhibitors such as vildagliptin, insulin secreting compounds and its biomimetics such as cinnamic acid and its derivatives, should be tested against *Acanthamoeba* cysts. Furthermore, the other major composite of cyst walls consists of acid-resistant proteins [10]. It is a cyst-specific protein of 21 kDa (CSP21) molecular weight, which is expressed in the process of encystment and absent in the trophozoite form of *A. castellanii* and its biosynthesis is regulated at the mRNA level [16]. The development in genetic engineering also provides a useful tool to study mechanistic target against *Acanthamoeba* cyst protein [16, 17]. For example, the development of anti-CSP21 antibodies is achieved to characterize *Acanthamoeba* taxonomy via specific binding to CSP21 [16, 17]. Behera et al. [18] carried out a more detailed protein analysis for both trophozoite and cyst forms of *Acanthamoeba* which suggests newer entities for targeting protein pathways. Based on MALDI-TOF/TOF analysis, two proteins from trophozoites were identified as hypothetical protein ACA1 and eukaryotic porin protein, while another pair of proteins were identified from cysts as chaperone protein DnaK and chaperonin protein, respectively. The exact functions of these proteins are not fully understood so far, but their anticipated roles are suggested to be as follows: eukaryotic porin proteins may have important role in the efflux of toxic metabolites and exudates during encystment and may also have an effect on pathogenicity. Similarly, proteins such as chaperone protein DnaK and chaperonin protein which belongs to heat shock proteins class, may have a role in folding of cyst specific proteins in cysts [18]. Hence, altering these genes’ expression may provide a means to control the life cycle of *Acanthamoeba*.

## Nanoparticles and antiacanthamoebic drugs

The recent decline in development of newer antimicrobial agents has contributed to increased disease burden as well as mortality rate and drug resistance [19]. As *Acanthamoeba* infections are considered rare, the development of antiacanthamoebic agents faces an even worse scenario when compared to other communicable diseases. The discovery of various antiacanthamoebic compounds tested in vitro has not been able to enter mainstream drug development due to their limited in vivo potential as well as general inefficacy against the cyst stage. A comprehensive review by our team [20] identifies and presents targets and mechanism of action of numerous drugs against *Acanthamoeba* although their in vivo efficacy remains undetermined. A few cases of re-purposing drugs are also reported to work well against *Acanthamoeba*, including some antibacterial agents: polymyxin B [21, 22], cefazolin [23], meropenem [24], moxifloxacin [25]; antifungal compounds: amphotericin B [26], azoles [27, 28]; and antineoplastic agents: alkylphosphocholines [29, 30]. Our studies also highlighted the use of re-purposed drugs against *Acanthamoeba* [31]. These included amiodarone, amlodipine, apomorphine, digoxin, haloperidol, loperamide, prochlorperazine, and procyclidine. Re-purposing of drugs has the advantage of an early start for new applications as they have been approved clinically for other diseases. Finding new sources of antiacanthamoebic drugs has also led several groups including ours to research natural products, and various plant extracts have been tested to exhibit cytotoxicity against *Acanthamoeba* [32–35].

**Fig. 3:**
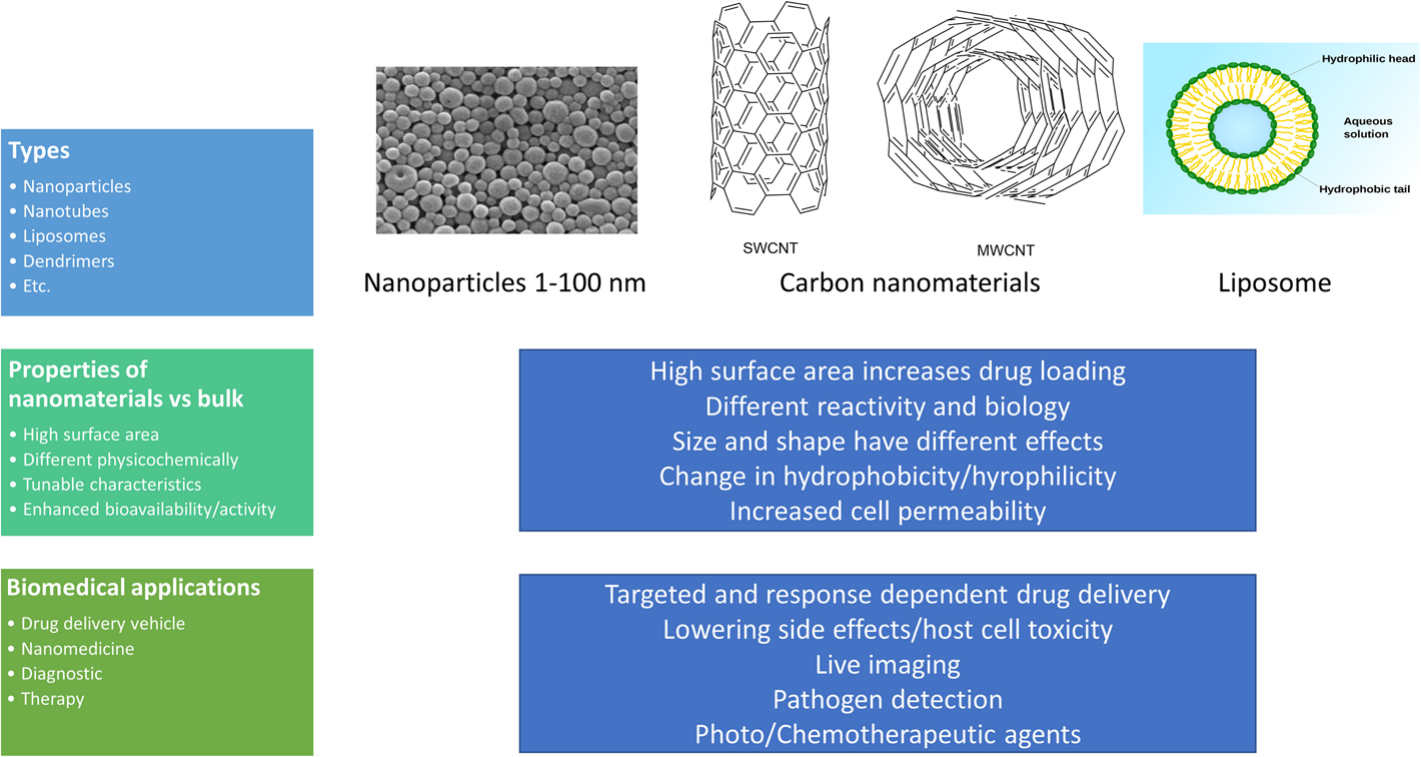
Types, examples, properties and biomedical applications of common nanomaterials. *Abbreviations*: nm, nanometer; SWCNT, single-walled carbon nanotube; MWCNT, multi-walled carbon nanotube

Nanotechnology is currently serving as a pivotal tool in different scientific ventures. Its applications in chemistry and biology have exceptionally emerged as potential source of novel solutions. The drastic contrast in chemical and biological properties of nanomaterials, as compared to their bulk counterparts, have pave their path in biomedicines for applications such as diagnosis, therapy, drug delivery etc. Nanomaterials can be simply described as materials and devices which lie within the range of nanometer scale [36]. The most utilized examples of nanomaterials are carbon nanotubes, nanoparticles, nanorods, liposomes etc. Nanomaterials have also recently been successfully employed extensively against numerous infectious diseases [37]. These are promising drug delivery systems due to their small size and cell penetration, and are associated with enhancing drug bioavailability and hence increasing their antimicrobial potency, reducing drug resistance by specifically targeting any cellular function, and increasing their stability in general (Fig. 3) [38]. The uses of nanomaterials against *Acanthamoeba* are so far limited to only a few examples. In our study liposomal complexation of pentamidine isethionate and ergosterol is shown to enhance its antiacanthamoebic activity [39]. In another report, silver nanoparticles have been successfully used to inhibit microbial growth and colonization on contact lenses, which shows the potential of nanoparticles in the development of contact lens solutions [40]. A formulation of nanoemulsion from plant extract of *Pterocaulon balansae* is used as a treatment of ocular AK. The extract is shown to contain high ratio of coumarin compounds which are naturally occurring plant products [35]. This report suggests that coumarin, polyphenols, and other natural products conjugated with nanomaterials hold promise against *Acanthamoeba* and possibly their cyst. The conjugation of chlorhexidine with gold nanoparticles has demonstrated a significant increase in its amoebicidal and cysticidal potency, with minimal associated host cell cytotoxicity by our group [41]. Metal oxide nanoparticles such as TiO_2_, Fe_2_O_3_, ZnO etc. are frequently used as antimicrobial agents due to their capability to generate reactive oxygen species (ROS) in the presence of UV light, which makes them an ideal candidate for application against microbes via photochemotherapy [42]. Based on these reports, nanomaterials, especially nanoparticles conjugated with natural products and broad spectrum antibacterial drugs, can be evaluated as a new generation of antimicrobial agents against *Acanthamoeba* infections. Furthermore, if constructed with cyst-targeting agents such as those discussed here, along with drug conjugation, they may produce effective nanomedicine formulations against *Acanthamoeba*. For example, if antimicrobial agents are coated on the surface of nanoparticles and those particles are fabricated with reagents having affinity with mannose-binding proteins, cellulose, galactose-polymer, polyhexamethylene biguanide (PHMB), neomycin, and other aforementioned compounds, this may result in enhanced overall antiacanthamoebic activity.

## Targeted antiacanthamoebic therapies

Targeting vehicles such as antibodies for a specific antigen, peptides enabling cells penetration and crossing barriers such as the blood-brain barrier (BBB), parasite specific biosynthetic pathways enzymes inhibitors, and mannose-conjugated antimicrobial agents, are important strategies for the targeted delivery of chemotherapeutic agents against *Acanthamoeba* infections. These avenues should provide tremendous advantages over non-specific therapies due to minimal side effects and host cells cytotoxicity. The targeted chemotherapy against *Acanthamoeba* has received limited attention, and only a few reports are published making it an open field for further research. For example, monoclonal IgA antibodies were shown to protect against AK, while the mode of protection was the inhibition of adhesion of *Acanthamoeba* to the corneal epithelium [43]. Drugs conjugated with parasite-specific adhesins can be of value in specific delivery of compounds. For example, mannose-binding protein on the surface of *Acanthamoeba* is an efficient target for delivery of mannose-conjugated drugs. A porphyrin molecule conjugated with mannose was used successfully in our study for the development of photodynamic therapy (PDT) against *Acanthamoeba* [44]. PDT is another promising technology which can be used to target resistant pathogens. The mode of PDT action is based on activation of photosensitizing compound with light of an appropriate wavelength to generate singlet oxygen and/or reactive oxygen species (ROS) which are known to induce cell death in the target pathogen. Targeted PDT is expected show high specificity towards the target cells and minimally toxic effects due to it not binding to host cells. However, such discrimination is sometimes difficult to achieve, hence the selective targeting of pathogenic *Acanthamoeba* remains a major concern. Additionally, more effective photosensitizers which can generate a burst of toxins at shorter pulsed interval of light will add practicality in their clinical applications. Hence, further work is needed to conjugate *Acanthamoeba* antibodies with more effective photoactivated compounds and/or drugs in the development of better antiacanthamoebic strategies. Another challenge in the successful treatment of amoebal brain infection is the inefficacy of drugs to cross the BBB to reach the site of infection and target the parasite. Cheng et al. [45] developed transactivator of transcription (TAT) peptide-modified gold nanoparticles, which are demonstrated to be capable of crossing the BBB and efficiently delivering drugs to brain tumour tissues [45]. These suggested strategies have a strong rationale, and if utilized smartly, could yield a breakthrough in treatment of infections due to *Acanthamoeba* as well as against other CNS pathogens. However, along with drug development, drugs administration also plays a significant role in the effectiveness of their performance, hence there is a desperate need to revisit the overall drug designing protocol against *Acanthamoeba* infections for efficient and more practical options.

## Conclusions

In summary, *Acanthamoeba* infections are complex biological disorders associated with very high rate of morbidity and mortality which require therapeutic strategies that recognize and respond to their dynamic nature. The molecular target-inspired approaches such as those presented above, in our opinion represent appealing frontiers for research and need to be evaluated further to develop more effective therapeutic options against *Acanthamoeba* as a whole and in particular against its cyst stage.
